# ChIPmentation for epigenomic analysis in fission yeast

**DOI:** 10.1007/978-1-0716-4168-2_18

**Published:** 2025-01-01

**Authors:** Felix Selasi Dewornu, Pin Tong, Sito Torres-Garcia, Alison Pidoux, Manu Shukla, Robin Allshire

**Affiliations:** 1 Wellcome Centre for Cell Biology and Institute of Cell Biology, School of Biological Sciences, The University of Edinburgh, Swann Building, King’s Buildings, Mayfield Road, Edinburgh EH9 3BF United Kingdom

**Keywords:** ChIPmentation, ChIP-Seq, Protein-DNA interactions, Nucleosome, Histone modifications, Heterochromatin, CENP-A^Cnp1^, Fission yeast

## Abstract

Histone modifications and transcription factor-DNA interactions regulate vital processes such as transcription, recombination, repair, and accurate chromosome segregation. Chromatin immunoprecipitation followed by sequencing (ChIP-Seq) has been instrumental in studying genome-wide association of DNA or chromatin associated factors and histone modifications. Here, we describe a ChIPmentation protocol adapted for fission yeast. This method merges Tn5 mediated tagmentation to existing ChIP protocols, resulting in lower sample input requirements with significant reduction in hands-on time and sample preparation costs.

## 1 Introduction

Chromatin Immunoprecipitation followed by sequencing (ChIP-Seq) has been instrumental in furthering our understanding of gene regulation and epigenetics by allowing mapping protein-DNA interactions in vivo (1–4). However, traditional ChIP techniques suffer from limitations such as extensive sample requirements and cumbersome protocols for sequencing library preparations (5, 6). To address these shortcomings, several new methodologies have emerged, offering improved efficiency, sensitivity, and reduced sample input requirements (7–12).

**Figure 1:**
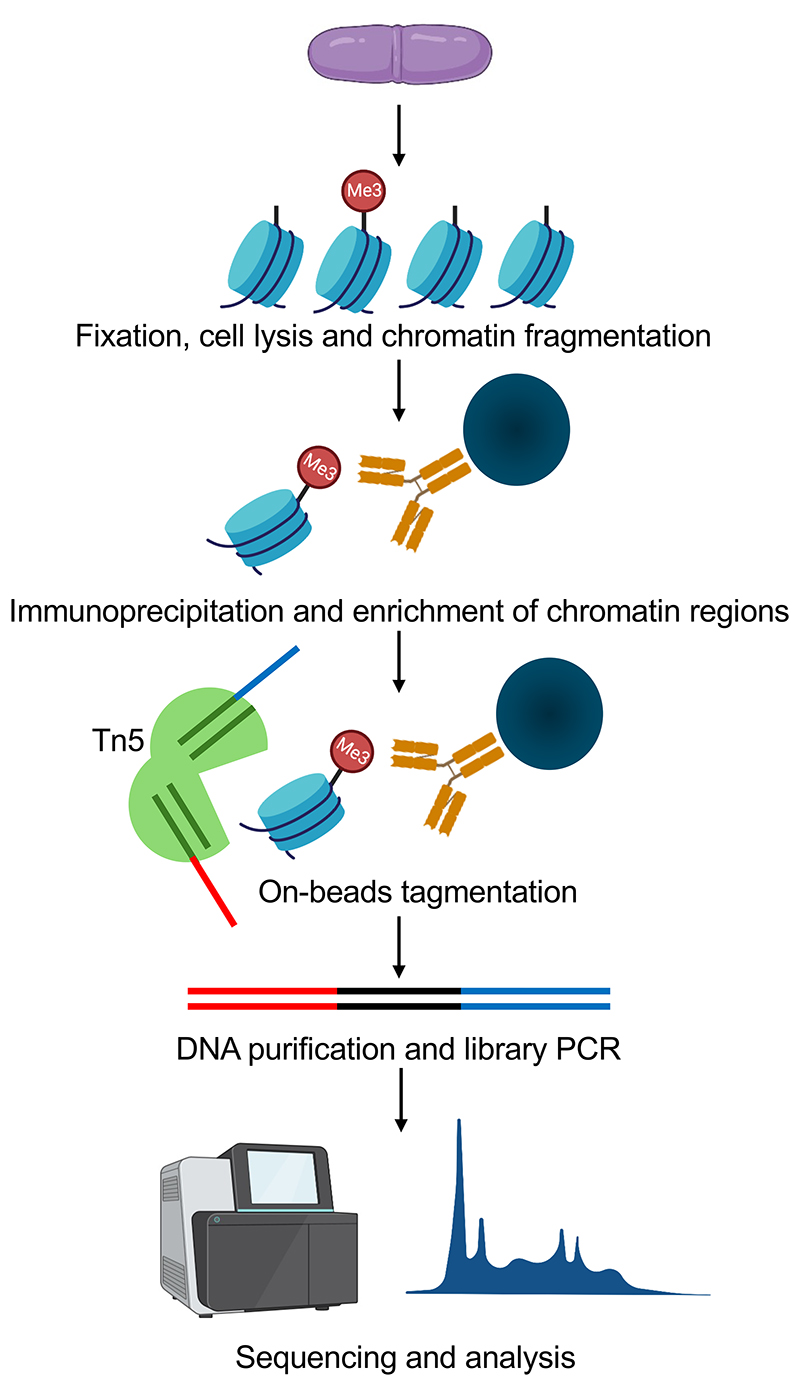
Schematic overview of ChIPmentation workflow in fission yeast. Cells are cross-linked, followed by lysis and immunoprecipitation. Tagmentation of enriched chromatin is performed using Tn5 transposase. Tagmented DNA is recovered and amplified for library preparation and subsequent sequencing. "Illustration was created using Biorender.com".

In this chapter, we describe the adaptation of ChIPmentation methodology, which integrates ChIP with transposase-based tagmentation (13, 14), for the fission yeast *Schizosaccharomyces pombe*. The initial experimental steps are identical to the standard ChIP procedure such as crosslinking, cell lysis and immunoprecipitation for enrichment of chromatin regions thus requiring minimal or no modifications to already established ChIP protocols for candidate protein or histone modifications. The key step is Tn5 transposase mediated tagmentation on immunoprecipitated chromatin immobilized on the beads. This obviates the need for DNA end polishing/repair, A tailing and adapter ligation steps, necessary for standard ChIP-Seq experimental pipelines. DNA recovered after tagmentation reaction is then directly amplified by PCR to add sequences required for next generation sequencing and multiplexing. A general schematic of the protocol is provided in Figure 1. ChIPmentation significantly reduces experimental time, cost, and sample requirements. By directly integrating sequencing adaptors during the immunoprecipitation step, this approach also minimizes DNA loss, enabling accurate genome-wide profiling of protein-DNA interactions at low cell numbers.

## 2 Materials

### 2.1 Equipment and Reagents

1. Fission yeast growth media such as Yeast Extract with Supplements (YES) or Pombe Minimal Glutamate (PMG) (15).
2. Spectrophotometer or hemocytometer to estimate cell numbers in cultures.
3. pH meter
4. 37% Formaldehyde solution (*see* Note 1)
5. 2.5 M Glycine
6. Phosphate Buffered Saline (PBS)
7. Protease inhibitor cocktail
8. 100 mM PMSF (*see* Note 1)
9. Acid washed glass beads (0.5 mm diameter)
10. Bead Beater
11. Lysis Buffer: 50 mM Hepes-KOH (pH7.5), 140 mM NaCl, 1 mM EDTA, 1% (v/v) Triton X-100, 0.1% (w/v) sodium deoxycholate, Protease inhibitors and 1 mM PMSF (Protease and PMSF added fresh). Store at 4°C.
12. Lysis Buffer with 0.5 M NaCl: Replace 140 mM NaCl in Lysis buffer with 500 mM NaCl). Store at 4°C.
13. Wash Buffer: 10 mM Tris-HCl (pH8), 0.25 M LiCl, 0.5% NP-40, 0.5% (w/v) Sodium Deoxycholate, 1 mM EDTA. Store at 4°C.
14. Tris wash buffer: 10 mM Tris-HCl (pH8.0).
15. 2X Tagmentation buffer: 20 mM Tris (pH 7.6), 10 mM MgCl2, 20% Dimethyl Formamide (*see* Note 1). Adjust pH of Tris buffer stock to 7.6 with 100% acetic acid).
16. Loaded Tn5 transposase
17. ChIPmentation Elution Buffer: 12.5 mM Tris-HCl (pH 8.0), 375 mM NaCl, 6.25 mM EDTA, 1.25% SDS.
18. Nuclease free molecular biology grade water
19. 20% SDS solution
20. Protein G Dynabeads
21. Anti H3K9Me2/3 and anti GFP antibodies
22. 10mg/ml Proteinase K solution
23. DNase Free RNaseA (0.5 mg/ml)
24. Thermomixer
25. Bioruptor sonicator
26. Screw capped, regular and DNA LoBind microcentrifuge tubes
27. Monarch PCR & DNA cleanup kit (or equivalent)
28. Absolute Ethanol
29. 10 mM Tris pH 8.0 or EB buffer
30. Phusion High-Fidelity DNA polymerase
31. PCR primers for Illumina sequencing
32. Thermocycler
33. Ampure XP beads
34. Qubit™ dsDNA-HS Assay kit
35. Qubit™ Fluorometer
36. DynaMag™-2 Magnet
37. Agilent 2100 Bioanalyzer system
38. High Sensitivity DNA Reagents kit
39. High Sensitivity DNA Chips
40. MiniSeq High throughput Reagent Kit (150-cycles)
41. MiniSeq

## 3 Method

### 3.1 Fixation of cells

1. For each ChIP grow 20 ml culture in YES or PMG as appropriate, ideally to 5 x 10^6^ cells/ml at 32°C, i.e. 10^8^ cells per ChIP. When doing more than one ChIP, scale up accordingly.
2. Transfer 20 ml cultures to 50 ml falcon tubes. Add formaldehyde to 1% final concentration to each culture (540 μl 37% formaldehyde per 20 ml). Swirl well to mix. Leave to fix at room temperature in a fume hood for 15 minutes.
3. Stop fixation by addition of 1/20th volume of 2.5 M glycine, mix by inverting the tubes and keep for 5 minutes at room temperature.
4. Spin for 1-2 min at 3500xg, 4°C in benchtop centrifuge. Pour off the supernatant.
5. Wash the cell pellet twice with 40 ml ice-cold PBS (resuspend pellet in a small volume first). Keep cells cold or on ice from this point onwards.
6. Transfer one ChIP equivalent of cells (10^8^ cells) to round-bottomed screw-capped tube in 1 ml PBS (*see* Note 2). Spin at 4°C in cooled microcentrifuge at ~4000xg. Remove supernatant carefully without disturbing the pellet. Freeze pellet on dry ice. Pellets can be stored at –80°C for several months.

### 3.2 Cell lysis, chromatin fragmentation and immunoprecipitation

1. Thaw cell pellets on ice. Add 300 μl ice-cold Lysis Buffer (protease inhibitors and PMSF are added fresh). Resuspend the cells and add 500 μl small glass beads (small microcentrifuge tube can be used as a scoop).
2. Put tubes into bead-beater, making sure that lids are tightened fully. Bead beat for 1 minute, twice with resting on ice for 2 minutes in between (*see* Note 3).
3. Heat needle (21-22 gauge) in Bunsen flame, quickly and carefully pierce bottom of screw-cap tube containing the lysate. Make three holes in each tube. Place this tube securely inside a labelled 1.5 ml microcentrifuge tube. Spin at very low speed e.g. at 1000 rpm for 30 seconds in cooled microcentrifuge. Do 6-8 tubes at a time.
4. Discard upper tube. Gently vortex the microcentrifuge tube containing collected lysate. There should be ~300 μl lysate.
5. Sonicate for 20-22 cycles on a 30 second on/off cycle on a Bioruptor at 4-5°C (*see* Note 4)
6. Centrifuge at 17000xg for 20 minutes at 4°C to pellet debris. Transfer supernatant to a fresh tube.
7. During the 20-minute centrifugation step, prepare Protein G Dynabeads. Use 15 μl of well mixed bead slurry per ChIP. Collect the beads by placing on a magnetic stand for 1-2 minutes and remove the supernatant. Resuspend beads in 1 ml ChIP lysis buffer by inverting the tube several times off the magnet. Collect the beads as before and repeat this process thrice. Upon removal of the supernatant from the last wash, resuspend the beads in the same volume of the lysis buffer as the volume of the beads taken at the start i.e. 15 μl per ChIP sample.
8. Save 10 μl of the lysate and transfer to a new tube. This would serve as the input.
9. To ~270 ul lysate add 1-3 μl antibody (depending upon the abundance of you protein of interest, usually ~1ug would a good starting point to ensure saturating conditions) and 15 μl washed Protein G Dynabeads. Incubate on a rotating wheel overnight at 4°C. Shorter incubation times of 3-4 hours can also be sufficient.
10. Collect beads by placing tubes on a magnetic stand and remove the supernatant. Wash with 0.9 ml of each of the following buffers:
  - Lysis Buffer – 10-minute wash
  - Lysis Buffer with 0.5 M NaCl – 10-minute wash
  - Wash Buffer – 10-minute wash
  - 10 mM Tris pH 8.0 – brief wash by inverting the tubes 15-20 times making sure that all the beads are resuspended.
11. After inverting the tubes at the last wash, briefly centrifuge the tubes at low speed (~1000 rpm) to collect all the liquid from the lids without pelleting the beads, transfer the resuspended beads to a fresh DNA LoBind tube. Collect the beads on a magnetic stand, remove all the supernatant and immediately proceed to the next step (*see* Note 5). From this point on, DNA low bind tubes should be used for the rest of the protocol.

### 3.3 Tagmentation and DNA recovery

1. Resuspend beads in 20 μl 1X tagmentation buffer containing 0.25-1 μl loaded Tn5, with 0.5 μl as a starting point (*see* Note 6). Optimise further, if needed, based on the fragment length distribution determined later through Bioanalyzer trace of your libraries. To 1.5 μl of Input, add 18.5 μl of 1X tagmentation buffer containing 0.25-0.5 μl loaded Tn5. Incubate all samples at 37°C for 30 minutes with intermittent mixing.
2. Add 80 μl of ChIPmentation Elution buffer to each sample to stop the reaction. Incubate samples on a shaking incubator (900-1000rpm) at 65°C for 4-6 hours (or overnight) for reverse crosslinking.
3. Cool samples down and add 2 μl DNase free RNaseA (0.5 μg/μl), incubate at 37°C for 1 hour on a thermomixer with shaking at ~900 rpm.
4. Add 2.5 μl of 10mg/ml Proteinase K and incubate at 55°C for 2 hours (with shaking) on a thermomixer.
5. Briefly spin the tubes to collect all the liquid at the bottom. Place IP tubes on a magnetic stand, collect supernatant and transfer to a new DNA LoBind tube.
6. Purify all the DNAs (IPs and Inputs) using a PCR and DNA clean up kit (*see* Note 7). Elute DNA in 25 μl volume of EB of the kit (*see* Note 8).

### 3.4 PCR amplification of tagmented DNA, clean up and library quality control

**Figure 2:**
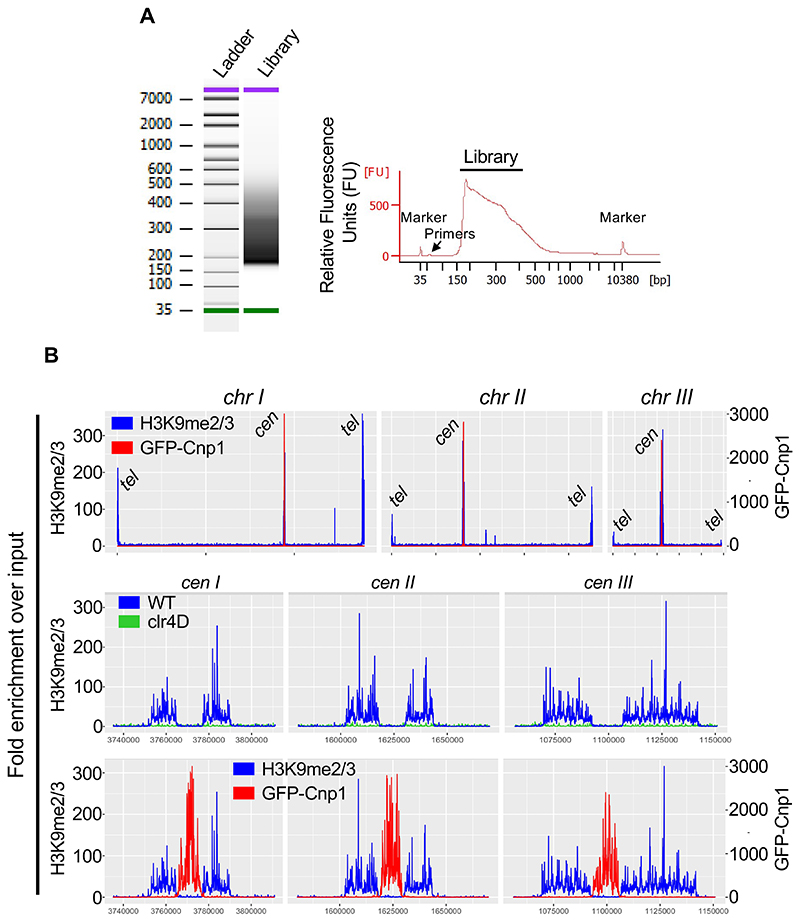
Effective detection of H3K9 methylation and CENP-A^Cnp1^ by ChIPmentation in fission yeast cells. **A**. Typical BioAnalyzer traces obtained from representative ChIPmentation experiment in *S. pombe*. Library fragment distributions and presence of primers are indicated. **B**. Representative profiles generated by ChIPmentation for H3K9 methylation and CENP-A^Cnp1^. Respective H3K9me2/3 (shown in blue) and CENP-A^Cnp1^ (shown in red) distributions across three chromosomes are shown in the upper panel. *clr4D* represents a negative control where the sole H3K9 methyltransferase gene *clr4*^*+*^ is deleted resulting in no heterochromatin present (shown in green, middle panel). An expanded view of centromere regions is shown in the lower panel for indicated datasets.

1. Set up the following PCR reaction on ice in a thin-wall 0.5 ml PCR tube, using a different barcode for each sample. Use primers as described in (16). Remaining DNA could be saved as a backup and stored at -20°C.
  10 μl adapter-ligated DNA
  10 μl 5x Phusion HF buffer
  1 μl 10 mM dNTPs
  2.5 μl 10 µM Universal or barcoded i5 primer
  2.5 μl 10 µM barcoded i7 primer
  1.5 μl DMSO
  0.5 μl Phusion polymerase
  22 μl ddH2O
2. Mix and quick spin. Place in thermocycler and begin cycling program with heated lid (*see* Notes 9 and 10).
  Cycle 1: 58°C for 5 minutes (gap filling step)
  Cycle 2: 72°C for 5 minutes (gap filling step)
  Cycle 3: 98°C for 30 seconds
  Cycle 4: 98°C for 20 seconds
  Cycle 5: 65°C for 20 seconds
  Cycle 6: 72°C for 10 seconds
  Repeat Cycles 4-6 10-14 times.
  72°C for 5 min and hold at 10°C
3. Do a 1:1.6 X Ampure XP bead cleanup (80 μl beads). Finally elute in 25-30 μl EB.
4. Check 1 μl library sample on Bioanalyzer using High Sensitivity DNA kit to assess library fragment size distribution and presence of adapters. See Figure 2A for typical library profiles. If primers are present in significant amounts, an additional 1:1.3 X Ampure XP bead clean up could be performed to remove the PCR primers.
5. Measure the library concentration on QubitTM fluorometer using the QubitTM dsDNA-HS Assay kit using 1-2 μl of purified libraries. Use the library average size information obtained from Bioanalyzer profiles in combination with library concentrations calculated from QubitTM fluorometer for multiplexing and creating the final library pool for sequencing.
6. Libraries can now be sequenced on all Illumina sequencers. Typically, five to ten million reads provide sufficient coverage for fission yeast ChIPmentation libraries.

### 3.5 Data analysis

1. Quality control analysis was run on paired-end reads of each sample (IP and Input) using FastQ Screen (v0.2.2) (17).
2. For each input and IP sample, reads were trimmed in pairs (forward and reverse) with Trim Galore (v0.3.2) (18) to remove sequencing adapters and improve read quality.
3. This was followed by another quality control analysis with FastQC (v0.11.2) to confirm trimming efficiency.
4. Validated paired reads were aligned the S. pombe reference genome (972h^-^, ASM294v2.22) using BWA (v0.7.17) (19). BAM files generated were sorted and indexed with Samtools (v1.9) (20). BamCoverage (deepTools v3.0) was used to generate coverage bigwig files normalized to BPM (21).
5. IP/input ratios were calculated with bamCompare (deepTools v3.0), normalized to BPM with bin size of 50. Enrichment plots were visualized with the Integrative Genomics Viewer (IGV) software (Figure 2B) (22).
6. The complete Snakemake (23) used for data analysis is available at https://github.com/SitoTorres/ChIPmentation_S.pombe_Snakemake/tree/main

## 4 Notes

1. Formaldehyde is toxic and a teratogen upon skin exposure, inhalation or ingestion. PMSF and DMF are toxic. Use appropriate safety gear such as a lab coat, eye protection and gloves. Use you institution specific containment and disposal routes for all the steps involving solutions containing these.
2. If more cells are collected for several ChIPs, cells can be aliquoted into ChIP sized aliquots in screw capped tubes at this point by resuspending the cells at 10^8^ cells/ml in PBS. Upon centrifugation and removal of PBS, the cell pellets can be stored at -80°C.
3. Bead beating time and cycles could be different on different homogenizers. Optimize the step accordingly. Overheating of the samples should be avoided as this could affect epitopes and negatively affect efficiency of immunoprecipitation.
4. Sonication efficiency could vary on different equipment. Optimize the sonication step to get DNA fragments in the range of 200-600 base pairs. This step should be optimized and checked before proceeding further with the protocol.
5. This step is performed to ensure that chromatin fragments bound non-specifically to microfuge surface during the immunoprecipitation step do not carry through to the next steps. Additionally, as the DNA amounts are expected to be very small after this stage, using DNA low binding tubes would help in better recovery of DNA fragments for subsequent steps.
6. In Addition, loaded Tn5 could made in house as described (14) and following double-stranded adapters with 19mer Tn5 mosaic ends can be used.
  Mosaic end_reverse [PHO]CTGTCTCTTATACACATCT
  Mosaic end_Adapter A TCGTCGGCAGCGTCAGATGTGTATAAGAGACAG
  Mosaic end_Adapter B GTCTCGTGGGCTCGGAGATGTGTATAAGAGACAG
7. DNA fragments at this stage are expected to be very small (100bp-300bp). Follow the recommendation for the sample to binding buffer ratio of the particular DNA clean up kit to ensure that smaller fragments are not lost.
8. 1-5 μl of samples (respective Inputs and IPs) from step 3.3.6 can be used to perform a qPCR to determine enrichment levels at this stage.
9. Alternative polymerases could be used for library amplification. The initial PCR cycles at 58°C and 72°C serve to fill the gaps left behind by tagmentation reaction. Thus, care should be taken when using a Hot Start DNA polymerase and PCR reactions should be heated to activate the DNA polymerase before addition of tagmented template DNA.
10. Do not add extra PCR cycles to amplify the signal seen by Bioanalyser (or Tapestation). Extra PCR cycles would reduce the complexity of the library and may result in very high level of PCR duplicates.
